# Hepatobiliary Manifestations of Inflammatory Bowel Disease

**DOI:** 10.1155/2000/98384

**Published:** 2000-08

**Authors:** Mohammed Iqbal Memon, Breda Memon, Muhammed Ashraf Memon

**Affiliations:** 1 Department of Community Health Guild NHS Trust and Lancashire Postgraduate Medical School Preston United Kingdom; 2 Department of Surgery Queen's Medical Center Nottingham United Kingdom; 3 Astley House Whitehall Road Darwen Lancashire BB3 2LH United Kingdom

## Abstract

Hepatobiliary manifestations occur quite frequently in patients suffering from chronic ulcerative colitis and Crohn's disease and carry with them considerable morbidity and mortality. Although the true incidence is difficult to determine, clinically, significant hepatobiliary disease occurs in 5%–10% of patients. At the present moment, the aetiology and pathogenesis of inflammatory bowel disease and its systemic manifestations remains speculative. For those hepatobiliary manifestations that respond to therapy of the underlying bowel disease, medical and/or surgical therapy must be aggressively pursued. More urgent research is required towards understanding the underlying cause(s) of the primary bowel disease and its systemic manifestations in order to improve the overall management of this condition.


*HPB Surgery*, 2000, Vol. 11, pp. 363-371
Reprints available directly from the publisher
Photocopying permitted by license only

© 2000 OPA (Overseas Publishers Association) N.V.
Published by license under
the Harwood Academic Publishers imprint,
part of The Gordon and Breach Publishing Group.
Printed in Malaysia.

### Hepatobiliary Manifestations of Inflammatory Bowel Disease

MOHAMMED IQBAL MEMON[a], BREDA MEMON[b] and MUHAMMED ASHRAF MEMON[c,*]

[a]*Department of Community Health, Guild NHS Trust and Lancashire Postgraduate Medical School, Preston UK;* [b]*Private Practice;* [c]*Department of Surgery, Queen's Medical Center, Nottingham, England, UK*

*(Received 21 November 1999)*

**Hepatobiliary manifestations occur quite frequently in patients suffering from chronic ulcerative colitis and Crohn's disease and carry with them considerable morbidity and mortality. Although the true incidence is difficult to determine, clinically significant hepatobiliary disease occurs in 5%–10% of patients. At the present moment, the aetiology and pathogenesis of inflammatory bowel disease and its systemic manifestations remains speculative. For those hepatobiliary manifestations that respond to therapy of the underlying bowel disease, medical and/or surgical therapy must be aggressively pursued. More urgent research is required towards understanding the underlying cause(s) of the primary bowel disease and its systemic manifestations in order to improve the overall management of this condition.**

*Keywords:* Inflammatory bowel disease, chronic ulcerative colitis, Crohn's disease, extraintestinal manifestations

### INTRODUCTION

Chronic ulcerative colitis (CUC) and Crohn's disease (CD), though chiefly effect the gastrointestinal tract, are frequently associated with a wide array of extraintestinal manifestations (EIM), the incidence of which varies between 25% and 36% [1-3]. Hepatobiliary manifestations are amongst the most common EIM associated with inflammatory bowel disease (IBD). They not only complicate the management of the primary disease but also contribute significantly to mortality and morbidity. Even before CUC was recognized as a clinical entity, fatty liver changes with diffuse colonic ulceration were described as early as 1874 by Thomas [4] which were confirmed by Lister in 1889 [5] who reported a patient with CUC and secondary diffuse hepatitis. Further evidence of association between CUC and hepatic involvement emerged from an autopsy study [6]. Although initial studies failed to show any association between CD and hepatobiliary disease, it soon became apparent that liver and biliary tract involvement occurs with equal frequency in patients with CD and CUC [7].

Although the prevalence of hepatobiliary disease in patients with IBD varies widely in different series from 2%-95%, clinically significant liver disease occurs in only 5%-10% of patients

*Address for correspondence: Astley House, Whitehall Road, Darwen, Lancashire BB3 2LH, England, UK. Tel.: +44 1254 760717, Fax: +44 1254 873671, e-mail: mmemon@yahoo.com

[8] (Tab. I). Such a discrepancy between the various series occurs because of a number of factors which include (a) on how aggressively diagnostic studies are pursued; (b) the number of patients included with mild, moderate and severe disease; (c) whether the disease was active or in remission and (d) the type of bowel involvement *i.e.*, extensive or limited. There is no consistent temporal relationship between the onset of symptoms and hepatobiliary abnormalities [7]. On average, CUC is present for approximately 8 years before the hepatic abnormalities become apparent [7]. The onset of symptoms may precede, follow or occur at the time of exacerbation of bowel disease. Associated hepatic conditions range from those which are little more than laboratory abnormalities, to those that are life-threatening. An interesting observation noted in patients with CD is, if the disease is restricted to the small bowel, hepatic involvement is rare.

Because the etiology and pathogenesis of IBD and the associated hepatobiliary complications remain elusive and speculative in the large part, it remains unresolved if these manifestations represent the systemic nature of the IBD or whether they truly are complications of the colitis.

### FATTY LIVER (HEPATIC STEATOSIS)

Fatty liver occurs in 15%–80% of patients with IBD [9]. Patients with severe colitis, fulminant first attack of colitis and those requiring colectomy over the age of 50 have the highest risk of developing this condition [8]. The incidence, however, falls to approximately 5% with mild disease [10]. Numerous factors may contribute towards this condition including malnutrition, bacterial metabolites, anemia, protein loss, drugs such as corticosteroids and tetracycline, chronic debilitating illness and total parental nutrition. Histological analysis reveals large fat droplets within hepatocytes with minimum surrounding inflammation. Fortunately, fatty liver does not progress to debilitating liver disease. This is an asymptomatic condition, although abdominal examination may reveal hepatomegaly. Treatment of the underlying bowel pathology and improving general health of the patients is all that is required. With advances in the treatment of IBD, the incidence of this condition may be far less than previously reported.

### CHOLELITHIASIS

Cholelithiasis occurs in 4%–34% of patients with IBD [2, 8, 11–13]. It occurs more frequently in CD than in CUC, and is perhaps more a secondary complication rather than a systemic manifestation. In CD, there is a positive correlation between stone formation and location, extent, and duration of ileal involvement as well as ileal resection and increased patient age [2, 13–15]. For CUC, total colitis extending to the caecum poses the greatest risk for gallstones

TABLE I Hepatobiliary manifestations of IBD

| | Chronic ulcerative colitis | Crohn's disease |
|---|---|---|
| More commonly associated (> 5%) | • Hepatic steatosis<br>• Small duct primary sclerosing cholangitis (Pericholangitis) | • Hepatic steatosis<br>• Cholelithiasis<br>• Hepatic amyloidosis |
| Less commonly associated (≤ 5%) | • Primary sclerosing cholangitis<br>• Bile duct carcinoma<br>• Cryptogenic cirrhosis<br>• Cholelithiasis | • Primary sclerosing cholangitis<br>• Autoimmune chronic active hepatitis<br>• Cryptogenic cirrhosis |
| Rare associations(< 1%) | • Hepatic amyloidosis<br>• Hepatic granuloma<br>• Pancreatitis<br>• Herpes simple hepatitis | • Hepatic granuloma<br>• Liver abscess<br>• Bile duct carcinoma |

[16]. The formation of cholesterol gallstones stems from the interruption of the normal enterohepatic circulation of bile acids. Disruption of the normal enterohepatic circulation results in depletion of the bile salt pool and the subsequent secretion of lithogenic bile [17,18]. The mechanism involved in the formation of radiopaque pigmented stones is not completely understood. If the gallstones becomes symptomatic, laparoscopic or open cholecystectomy is the treatment of choice.

### CHOLANGIOCARCINOMA/ HEPATOCELLULAR CARCINOMA

Cholangiocarcinoma in association with CUC was first recognized by Parker and Kendall in 1954 [19]. Since then this relationship between biliary tract tumors and CUC has been well established. The reported incidence of cholangiocarcinoma in patients with IBD is between 0.4% – 1.4%, which is 10 – 100 times greater than reported for the general population [9,20–24]. On the other hand, the incidence of CUC in patients with bile duct cancer is between 6% and 14% [24]. It is more common in men and occurs in the fourth and fifth decades, about 20 years earlier than in the general population [8,10,14]. It occurs predominantly in patients with CUC, but has also been reported in patients with CD. Most cholangiocarcinomas develop in patients with preexisting PSC [25]. The greatest risk appears to be for those CUC patients with pancolitis and for those with an average duration of 15 years of disease [9]. There is no apparent relationship between the development of cholangiocarcinoma and the activity of bowel disease as it may develop during prolonged remission and even following proctocolectomy [21,22,26,27]. The clinical presentation of cholangiocarcinoma is that of progressive cholestatic jaundice. Radiographically, these tumors may present as polypoid or papillary masses, as rapidly progressive strictures, or as annular constricting lesions with proximal bile duct dilatation. Common sites of involvement are large biliary ducts or the bifurcation of the intrahepatic ducts. Endoscopic retrograde cholangiopancreatography (ERCP) or percutaneous transhepatic cholangiography (PTC) will demonstrate the majority of these lesions, while laparotomy may be reserved where diagnosis is in doubt. A retrospective study suggests that the measurement of tumor associated antigen CA 19–9, may be promising for the detection of cholangiocarcinoma complicating PSC [28]. The tumour usually pursues a progressive course and prognosis is very poor, with a median survival of 5 months [29]. Palliative surgery is the most effective treatment but has little impact on prolonging the survival [8]. Similarly the results of orthotopic liver transplantation has been disappointing due to early recurrences [30].

Fibrolamellar hepatocellular carcinoma has recently been diagnosed in two patients with CUC and PSC [31,32]. Both of these patients were free of cirrhosis. One of these patients received liver transplantation but died of tumor recurrence [32].

### LIVER CIRRHOSIS

Cryptogenic liver cirrhosis occurs in approximately 1 – 5% of patients with hepatic abnormalities and IBD [33,34]. It is more frequently seen in extensive ulcerative colitis or Crohn's colitis as compared to Crohn's ileitis. Autoimmune Chronic Active Hepatitis (AICAH), pericholangitis and PSC are important risk factors [9]. In some patients who have received multiple blood transfusions, hepatitis C may be a causative agent. The treatment of choice for end stage liver disease is liver transplantation. Central portosystemic shunts must be avoided as not only do they increase perioperative mortality rates but they also make liver transplantation technically more challenging [8]. Patients may present with signs and symptoms of end-stage liver disease such as jaundice, ascites, encephalopathy, spontaneous bacterial peritonitis and gastric and oesophageal bleeding. Oesophageal variceal bleeding can easily be treated using endoscopic banding, sclerotherapy or transjugular intrahepatic portosystemic shunting (TIPS). The majority of authors report that neither medical or surgical treatment of IBD has any effect on the natural history of cirrhosis [35]. Eade *et al.* [36] nonetheless did show arrest or regression of fibrosis in the majority of patients after colectomy.

### AUTOIMMUNE CHRONIC ACTIVE HEPATITIS (AICAH)

Autoimmune chronic active hepatitis occurs in 1% of cases of IBD, mainly in CUC [3,37]. Conversely, the incidence of IBD in patients with AICAH varies between 4% and 30% [35]. There is no relation between the activity of the hepatitis and the severity or activity of colitis. Olsson and Hulten [38], however, have reported significant improvement in AICAH following colectomy. Factors implicated in the causation of AICAH include blood transfusion, ethanol abuse, PSC and autoimmune phenomenon with genetic predisposition [9]. Progression to post-necrotic cirrhosis may occur. There is some evidence that patients with severe AICAH with CUC respond less favourably to treatment compared to their counterparts without CUC [39].

### HEPATIC AMYLOIDOSIS

Hepatic amyloidosis occurs in less than 1% of patients with IBD, and the majority of these patients have CD [8,10,14]. There is no relationship between the site of bowel involvement and occurrence of amyloidosis [40]. It usually involves the media of the branches of the hepatic artery in the portal triad and, to a lesser extent, the portal venules and bile ductules [40,41]. Clinically patients may present with hepatomegaly. Regression has been reported following the resection of inflamed bowel [42,43]. These patients may also develop renal amyloidosis resulting in renal impairment which may culminate into renal failure in the post-operative period [9]. The prognosis of these patients is generally poor.

### HEPATIC ABSCESS

Pyogenic liver abscess occurs in 0.3% of patients with CD. The abscesses are frequently multiple and carry a high mortality [2]. A number of mechanisms have been proposed including (a) seeding from the portal vein; (b) direct extension from intra-abdominal abscesses; (c) indirect complications from CD such as acalculous cholecystitis or enteric fistulas; and (d) from sepsis occurring in malignancies metastasizing to the liver [8,9]. Patients may present with right upper quadrant pain, pyrexia, nausea, vomiting, hepatomegaly, jaundice, and right subcostal tenderness. The diagnosis can be made on the basis of history, clinical examination, cultures, ultrasonography, computerized tomography (CT), magnetic resonance imaging (MRI) and radionuclide scanning [9]. Streptococcus, Klebsiella, and E. Coli are the three most common organisms identified and treatment includes broad spectrum antibiotics combined with percutaneous drainage under ultrasound or CT guidance. Surgical drainage is reserved for patients who deteriorate or who do not respond to the above regimen within two weeks.

### HEPATIC GRANULOMAS

Hepatic granulomas are rare findings occurring commonly in patients with CD, although they have been described in patients with CUC. Usually asymptomatic, they may present with fever, hepatomegaly and jaundice. They may cause modest elevation in serum alkaline phosphatase (50% of cases) and may resolve when the diseased bowel is resected [33,44,45].

### PRIMARY SCLEROSING CHOLANGITIS

Primary Sclerosing Cholangitis (PSC) is a chronic, slowly progressive, cholestatic liver disease of unknown pathology, most commonly occurring in young men between the ages of 20 and 40 years [46,47]. It is characterized by progressive chronic stenosing and fibrosing inflammation of both the intrahepatic and extrahepatic biliary tree. Generally accepted diagnostic criteria for PSC are outlined in Table II. Primary sclerosing cholangitis occurs in 4%–10% of patients with CUC [9,48,49] and 3.4% of patients with CD [50]. However, when CD involves the large bowel, the incidence of PSC increases to 9%, a rate similar to CUC [51]. On the other hand between 54% and 100% of PSC patients have IBD [47,52–56].

Currently the etiopathogenesis of both PSC and IBD remains speculative. Present evidence however suggests that PSC is an autoimmune disorder, where immunologic factors triggered by a virus or bacteria in genetically susceptible individuals are thought to damage bile duct epithelial cells [57]. Other factors that have been implicated in the etiology of PSC include environmental toxins, hepatic copper, viral infections (hepatitis A,B,C,D, cytomegalovirus, and reovirus), portal bacteremia, absorbed colonic toxins, toxic bile acids, genetic predisposition (HLA-B8, HLA-DR2, HLA-DR3 and HLA-DRw52A), ischemic arteriolar injury and altered cellular and humoral immunological responses [47,58–66].

There is no relationship between PSC and the onset, duration, activity, or extent of CUC [2,67–69]. It can even present years after proctocolectomy [67–70]. Although most patients have no hepatobiliary symptoms or signs during the early phase of the disease, others will present with malaise, fatigue, jaundice, weight loss, right upper quadrant abdominal pain, hepatomegaly, pruritus, acute cholangitis and/or portal hypertension. Diagnosis is based on history, laboratory investigations, ERCP or PTC and liver biopsy. To date there is no effective treatment available which can reverse or halt the progression of PSC. In desperation there has been a surge of interest in the use of ursodeoxycholic acid in PSC [47,71]. Ursodeoxycholic acid (UDCA) has been investigated on the grounds that it: (a) is minimally toxic, (b) replaces the bile acid pool with a less toxic bile (compared to lithocholic acid), (c) decreases the expression of class I antigens on the biliary epithelium, thereby modifying immunological responses and (d) improves biochemical indices as well as histopathological features. In two randomized trials [72,73], however, the use of UDCA failed to show any improvement in clinical parameters, histology, or time to treatment failure or liver transplantation. Symptomatic treatments for PSC include cholestyramine, UDCA or/and antihistamines for pruritus, replacement of fat soluble vitamins (A,D,E,K), calcium and vitamin D for metabolic bone disease, ERCP, endoscopic sphincterotomy and stone extraction for obstructed juandice and cholangitis secondary to choledocholithiasis, antibiotics for bacterial cholangitis, endoscopic dilatations and stents for bile duct strictures, and cholecystectomy for gallstones.

A number of other therapies and strategies are also relevant to IBD complicated by PSC. First of all, the mainstay for end stage liver disease in a selected group of patients is liver transplantation. In the event of variceal bleeding, most

TABLE II Criteria for diagnosing PSC

- Cholestatic biochemical profile *i.e.*, alkaline phosphatase level greater than 1.5 fold over the normal limits for 6 months or more
- Generalized beading, stricturing or irregularity of the biliary system based on cholangiography
- Interlobular and septal bile duct fibrosis and obliteration on liver biopsy in the absence of other causes of chronic liver disease
- Exclude other cause of liver disease such as biliary calculi, biliary tract surgery, congenital biliary conditions, AIDS associated cholangiopathy, ischaemic stricturing, biliary neoplasms, chemical hepatitis, PBS or CAH

surgeons do not recommend hepatobiliary shunt surgery because it increases the risk of bacterial cholangitis and may increase the perioperative mortality of a potential liver transplantation [37]. Sclerotherapy is considered a treatment of choice in these patients. If variceal sclerotherapy fails to control the bleeding, transjugular intrahepatic portosystemic shunt (TIPS) should be considered as a bridge to liver transplantation [8]. Liver transplantation is ultimately recommended for patients with variceal bleeding or known varices with hypersplenism, rising serum bilirubin levels, decreased synthetic liver function, recurrent cholangitis, repeated radiological or endoscopic procedures to maintain the ductal patency or spontaneous bacterial peritonitis. Parastomal bleeding can be another troublesome source of problems in these patients after colectomy and ileostomy [74]. The complication of parastomal bleeding can be prevented by performing an ileoanal anastomosis, or ileal pouch anal anastomosis rather than fashioning an ileal stoma, in patients undergoing panproctocolectomy for IBD in the presence of PSC [74–76]. Primary sclerosing cholangitis seems to be an additional risk factor for the development of colon cancer in those with long standing CUC [72,77]. If carcinoma or precancerous lesions develop in the colon, a proctocolectomy is indicated.

### PERICHOLANGITIS OR SMALL DUCT PRIMARY SCLEROSING CHOLANGITIS

Pericholangitis or small duct primary sclerosing cholangitis is a subset of PSC which is diagnosed on the basis of liver biopsy in the presence of a normal cholangiogram. It occurs in 30% of patients with IBD, is usually benign, and its course often parallels the bowel disease activity and severity [33,78]. It is now believed that pericholangitis represents a continuum in the spectrum of PSC [79]. The majority of cases resolve with residual mild periductal fibrosis, some may progress to a chronic phase or to PSC or to cirrhosis [7,79,80].

### CONCLUSION

Hepatobiliary manifestations are an important cause of morbidity and mortality in patients with IBD. On one hand the presence of some of these manifestations may provide a justification for bowel resection, but on the other hand their presence may predict a complex and complicated perioperative recovery. Although some hepatobiliary complications are obviously directly related to local disease complications, such as stone formation or liver abscesses, or related to therapeutic side-effects, such as drug-induced liver steatosis, others appear to be systemically-mediated. Frustratingly, the aetiology and pathogenesis of IBD and its systemic manifestations including hepatobiliary disease remains mysterious. For now, we must settle for a more practical approach to understanding the relationship between hepatobiliary manifestations and bowel disease activity if and when it exists (Tab. III). For those hepatobiliary manifestations that respond to therapy of the underlying bowel disease, medical and/or surgical

TABLE III Hepatobiliary manifestations of IBD and their relationship to bowel activity and bowel surgery

| Hepatobiliary manifestations | Relationship to bowel activity | Relationship to bowel surgery |
|---|---|---|
| Hepatic Steatosis | Usually parallels* | May resolve* |
| Cholelithiasis | Unrelated | May deteriorate* |
| Pericholangitis | Unrelated | No change |
| Primary sclerosing cholangitis | Unrelated | No change |
| Autoimmune chronic active hepatitis | Unrelated | May improve* |
| Cryptogenic cirrhosis | Unrelated | No change |
| Adenocarcinoma of bile ducts | Unrelated | No change |
| Hepatic amyloidosis | Unrelated | May resolve* |
| Hepatic granulomas | Unrelated | May resolve* |

therapy must be aggressively pursued. The response of a given hepatobiliary manifestation to surgery at least provides a framework for considering the role of the surgeon in the management of these often difficult clinical problems.

### *References*

[1] Rankin, G. B. (1990). Extraintestinal and systemic manifestations of inflammatory bowel disease. *Med. Clin. North Am.*, **74**, 39–50.

[2] Greenstein, A. J., Janowitz, H. D. and Sachar, D. B. (1976). The extra-intestinal complications of Crohn's disease and ulcerative colitis: a study of 700 patients. *Medicine*, **55**, 401–412.

[3] Danzi, J. T. (1988). Extraintestinal manifestations of idiopathic inflammatory bowel disease. *Arch. Intern. Med.*, **148**, 297–302.

[4] Thomas, G. H. (1874). Ulceration of the colon with enlarged fatty liver. Transactions of the Pathology Society, *Philadelphia*, **4**, 87–93.

[5] Lister, J. D., *A specimen of the diffuse ulcerative colitis with secondary diffuse hepatitis*, Transactions of the Pathology Society of London, 1899, **50**, 130–135.

[6] Pollard, H. M. and Block, M. (1948). Association of hepatic insufficiency with chronic ulcerative colitis. *Arch. Intern. Med.*, **82**, 159–174.

[7] Holdstock, G., Iredale, J., Millward-Sadler, G. H. and Wright, R., Hepatic Changes in systemic disease. In: Millward-Sadler, G. H., Wright, R. and Arthur, M. J. P. (3rd edn.), *Wright's Liver and Biliary Disease: Pathophysiology*, Diagnosis and Management, London, WB Saunders Co. Ltd., 1992, pp. 995–1038.

[8] White, H. and Peters, M., Hepatobiliary disorders in inflammatory bowel disease. In: MacDermott, R. P. and Stenson, W. F. Eds., *Inflammatory Bowel Disease*, New York: Elsevier, 1992, pp. 405–417.

[9] Memon, M. A. and Nelson, H. (1996). Extraintestinal manifestations of inflammatory bowel disease. *Colon. Rectal. Surg.*, **9**, 1–29.

[10] van Erpecum, K. J. and van Berge Henegouwen, G. P. (1989). Hepatobiliary abnormalities in inflammatory bowel disease. *Netherlands J. Med.*, **35** (Suppl. 1) S.40–49.

[11] Heaton, K. W. and Read, A. E. (1969). Gall stones in patients with disorders of the terminal ileum and disturbed bile salt metabolism. *BMJ*, **3**, 494–496.

[12] Cohen, S., Kpplan, M., Gottlieb, L. and Patterson, J. (1971). Liver disease and gallstones in regional enteritis. *Gastroenterol*, **60**, 237–245.

[13] Baker, A. L., Kaplan, M. M., Norton, R. A. and Patterson, J. F. (1974). Gallstones in inflammatory bowel disease. *Am. J. Dig. Dis.*, **19**, 109–112.

[14] Williams, S. M. and Harned, R. K. (1987). Hepatobiliary complications of inflammatory bowel disease. *Radiol. Clin. North. Am.*, **25**, 175–188.

[15] Hill, G. L., Mair, W. S. and Goligher, J. C. (1975). Gallstones after ileostomy and ileal resection. *Gut*, **16**, 932–936.

[16] Lorusso, D., Leo, S., Mossa, A., Misciagna, G. and Guerra, V. (1990). Cholelithiasis in inflammatory bowel disease. A case-control study. *Dis. Colon. Rectum*, **33**, 791–794.

[17] Dowling, R. H., Bell, G. D. and White, J. (1972). Lithogenic bile in patients with ileal dysfunction. *Gut.*, **13**, 415–420.

[18] Vlahcevic, Z. R., Bell, C. C. Jr., Buhac, I., Farrar, J. T. and Swell, L. (1970). Diminished bile acid pool size in patients with gallstones. *Gastroenterol*, **59**, 165–173.

[19] Parker, R. G. F. and Kendall, E. J. C. (1954). The liver in ulcerative colitis. *BMJ*, **2**, 1030–1033.

[20] Akwari, O. E., van Heerden, J. A., Adson, M. A., Foulk, W. T. and Baggenstoss, A. H. (1976). Bile duct carcinoma associated with ulcerative colitis. *Rev. Surg.*, **33**, 289–293.

[21] Converse, C. F., Reagan, J. W. and DeCosse, J. J. (1971). Ulcerative colitis and carcinoma of the bile ducts. *Am. J. Surg.*, **121**, 39–45.

[22] Mir-Madjlessi, S. H., Farmer, R. G. and Sivak, M. V. Jr. (1987). Bile duct carcinoma in patients with ulcerative colitis. Relationship to sclerosing cholangitis: report of six cases and review of the literature. *Dig. Dis. Sci.*, **32**, 145–154.

[23] Morowitz, D. A., Glagov, S., Dordal, E. and Kirsner, J. B. (1971). Carcinoma of the biliary tract complicating chronic ulcerative colitis. *Cancer*, **27**, 356–361.

[24] Ross, A. P. and Braasch, J. W. (1973). Ulcerative colitis and carcinoma of the proximal bile ducts. *Gut*, **14**, 94–97.

[25] Wee, A., Ludwig, J., Coffey, R. J. Jr., LaRusso, N. F. and Wiesner, R. H. (1985). Hepatobiliary carcinoma associated with primary sclerosing cholangitis and chronic ulcerative colitis. *Hum. Pathol.*, **16**, 719–726.

[26] Ritchie, J. K., Allan, R. N., Macartney, J., Thompson, H., Hawley, P. R. and Cooke, W. T. (1974). Biliary tract carcinoma associated with ulcerative colitis. *QJM*, **43**, 263–279.

[27] Williams, S. M. and Harned, R. K. (1981). Bile duct carcinoma associated with chronic ulcerative colitis. *Dis. Colon. Rectum.*, **24**, 42–44.

[28] Nichols, J. C., Gores, G. J., LaRusso, N. F., Wiesner, R. H., Nagorney, D. M. and Ritts, R. E. Jr. (1993). Diagnostic role of serum CA 19–9 for cholangiocarcinoma in patients with primary sclerosing cholangitis. *Mayo. Clin. Proc.*, **68**, 874–879.

[29] Chapman, R. W. and Angus, P. W., The effect of gastroentistinal diseases on the liver and biliary tract. In: Bircher, J., Benhamou, J.-P., McIntyre, N., Rizzetto, M. and Rodes, J. (2nd edn.), *Oxford Textbook of Clinical Hepatology*, Oxford, Oxford University Press, 1999: pp. 1685–1691.

[30] Iwatsuki, S., Gordon, R. D., Shaw, B. W. Jr. and Starzl, T. E. (1985). Role of liver transplantation in cancer therapy. *Ann. Surg.*, **202**, 401–407.

[31] Snook, J. A., Kelly, P., Chapman, R. W. and Jewell, D. P. (1989). Fibrolamellar hepatocellular carcinoma complicating ulcerative colitis with primary sclerosing cholangitis. *Gut*, **30**, 243–245.

[32] Klompmaker, I. J., de Bruijn, K. M., Gouw, A. H., Bams, J. I. and Slooff, M. J. (1988). Recurrence of hepatocellular carcinoma after liver retransplantation. *BMJ Clin. Res. Ed.*, **296**, 1445.

[33] Dordal, E., Glagov, S. and Kirsner, J. B. (1967). Hepatic lesions in chronic inflammatory bowel disease. I. Clinical correlations with liver biopsy diagnoses in 103 patients. *Gastroenterol*, **52**, 239–53.

[34] Schrumpf, E., Fausa, O., Elgjo, K. and Kolmannskog, F. (1988). Hepatobiliary complications of inflammatory bowel disease. *Semin. Liver. Dis.*, **8**, 201–209.

[35] Harmatz, A. (1994). Hepatobiliary manifestations of inflammatory bowel disease. *Med. Clin. North Am.*, **78**, 1387–1398.
[36] Eade, M. N., Cooke, W. T. and Brooke, B. N. (1970). Liver disease in ulcerative colitis. *Lancet*, **2**, 718.
[37] O'Brein, J. Extraintestinal manifestations of inflammatory bowel disease. In: MacDermott, R. P. and Stenson, W. F. Eds., *Inflammatory Bowel Disease*, New York: Elsevier, 1992, pp. 387–404.
[38] Olsson, R. and Hulten, L. (1975). Concurrence of ulcerative colitis and chronic active hepatitis, Clinical courses and results of colectomy. *Scand J. Gastroenterol*, **10**, 331–335.
[39] Perdigoto, R., Carpenter, H. A. and Czaja, A. J. (1992). Frequency and significance of chronic ulcerative colitis in severe corticosteroid-treated autoimmune hepatitis. *J. of Hepatol.*, **14**, 325–331.
[40] Shorvon, P. J. (1977). Amyloidosis and inflammatory bowel disease. *Am. J. Dig. Dis.*, **22**, 209–213.
[41] Glenner, G. G. (1980). Amyloid deposits and amyloidosis: the beta-fibrilloses (second of two parts). *NEJM*, **302**, 1333–1343.
[42] Fausa, O., Nygaard, K. and Elgjo, K. (1977). Amyloidosis and Crohn's disease. *Scand J. Gastroenterol*, **12**, 657–662.
[43] Fitchen, J. H. (1975). Amyloidosis and granulomatous ileocolitis. Regression after surgical removal of the involved bowel. *NEJM*, **292**, 352–353.
[44] Mauer, H. L., Hughes, R. W., Folley, J. H. and Mosenthal, W. T. (1967). Granulomatous hepatitis associated with regional enteritis. *Gastroenterol*, **53**, 301–305.
[45] Eade, M. N. (1970). Liver disease in ulcerative colitis. I. Analysis of operative liver biopsy in 138 consecutive patients having colectomy. *Ann. Intern. Med.*, **72**, 475–487.
[46] Lee, Y. M. and Kaplan, M. M. (1995). Primary sclerosing cholangitis. *NEJM*, **332**, 924–933.
[47] Wiesner, R. H. (1994). Current concepts in primary sclerosing cholangitis. [Review] [108 Refs.] *Mayo. Clin. Proc.*, **69**, 969–982.
[48] Schrumpf, E., Fausa, O., Kolmannskog, F., Elgjo, K., Ritland, S. and Gjone, E. (1982). Sclerosing cholangitis in ulcerative colitis. A follow-up study. *Scand J. Gastroenterol*, **17**, 33–39.
[49] Shepherd, H. A., Selby, W. S., Chapman, R. W., Nolan, D., Barbatis, C., McGee, J. O. and Jewell, D. P. (1983). Ulcerative colitis and persistent liver dysfunction. *QJM*, **52**, 503–513.
[50] Raj, V. and Lichtenstein, D. R. (1999). Hepatobiliary manifestations of inflammatory bowel disease. *Gastroenterol Clin. North Am.*, **28**, 491–513.
[51] Rasmussen, H. H., Fallingborg, J. F., Mortensen, P. B., Vyberg, M., Tage-Jensen, U. and Rasmussen, S. N. (1977). Hepatobiliary dysfunction and primary sclerosing cholangitis in patients with Crohn's disease. *Scand J. Gastroenterol*, **32**, 604–610.
[52] Aadland, E., Schrumpf, E., Fausa, O., Elgjo, K., Heilo, A., Aakhus, T. and Gjone, E. (1987). Primary sclerosing cholangitis: a long-term follow-up study. *Scand J Gastroenterol*, **22**, 655–664.
[53] Chapman, R. W., Arborgh, B. A., Rhodes, J. M., Summerfield, J. A., Dick, R., Scheuer, P. J. and Sherlock, S. (1980). Primary sclerosing cholangitis: a review of its clinical features, cholangiography, and hepatic histology. *Gut*, **21**, 870–877.
[54] Fausa, O., Schrumpf, E. and Elgjo, K. (1989). Inflammatory bowel disease occurs in almost all patients with primary sclerosing cholangitis. *Scand J Gastroenterol*, **24** (Suppl. 159), 53 (Abstract).
[55] Lebovics, E., Palmer, M., Woo, J. and Schaffner, F. (1987). Outcome of primary sclerosing cholangitis. Analysis of long-term observation of 38 patients. *Arch. Intern. Med.*, **147**, 729–731.
[56] Sivak, M. V. Jr., Farmer, R. G. and Lalli, A. F. (1981). Sclerosing cholangitis: its increasing frequency of recognition and association with inflammatory bowel disease. *J. Clin. Gastroenterol*, **3**, 261–266.
[57] Nelson, H. (1990). Immunology of chronic ulcerative colitis. *Semin. Colon. Rectal. Surg.*, **1**, 147–157.
[58] Chapman, R. W., Varghese, Z., Gaul, R., Patel, G., Kokinon, N. and Sherlock, S. (1983). Association of primary sclerosing cholangitis with HLA-B8. *Gut*, **24**, 38–41.
[59] Donaldson, P. T., Farrant, J. M., Wilkinson, M. L., Hayllar, K., Portmann, B. C. and Williams, R. (1991). Dual association of HLA, DR2 and DR3 with primary sclerosing cholangitis. *Hepatol.*, **13**, 129–133.
[60] Prochazka, E. J., Terasaki, P. I., Park, M. S., Goldstein, L. I. and Busuttil, R. W. (1990). Association of primary sclerosing cholangitis with HLA-DRw52a. *NEJM*, **322**, 1842–1844.
[61] Holzbach, R. T., Marsh, M. E., Freedman, M. R., Fazio, V. W., Lavery, I. and Jagelman, D. A. (1980). Portal vein bile acids in patients with severe inflammatory bowel disease. *Gut*, **21**, 428–435.
[62] Palmer, K. R., Duerden, B. I. and Holdsworth, C. D. (1980). Bacteriological and endotoxin studies in cases of ulcerative colitis submitted to surgery. *Gut*, **21**, 851–854.
[63] Chapman, R. W. and Jewell, D. P. (1985). Primary sclerosing cholangitis–an immunologically mediated disease?. *West J. Med.*, **143**, 193–195.
[64] Das, K. M., Squillante, L., Chitayet, D. and Kalousek, D. K. (1992). Simultaneous appearance of a unique common epitope in fetal colon, skin, and biliary epithelial cells. A possible link for extracolonic manifestations in ulcerative colitis. *J. Clin. Gastroenterol*, **15**, 311–316.
[65] Bodenheimer, H. C. Jr., LaRusso, N. F., Thayer, W. R. Jr., Charland, C., Staples, P. J. and Ludwig, J. (1983). Elevated circulating immune complexes in primary sclerosing cholangitis. *Hepatol.*, **3**, 150–154.
[66] Lindor, K. D., Wiesner, R. H., Katzmann, J. A., LaRusso, N. F. and Beaver, S. J. (1987). Lymphocyte subsets in primary sclerosing cholangitis. *Dig. Dis. Sci.*, **32**, 720–725.
[67] Cangemi, J. R., Wiesner, R. H., Beaver, S. J., Ludwig, J., MacCarty, R. L., Dozois, R. R., Zinsmeister, A. R. and LaRusso, N. F. (1989). Effect of proctocolectomy for chronic ulcerative colitis on the natural history of primary sclerosing cholangitis. *Gastroenterol*, **96**, 790–794.
[68] Steckman, M., Drossman, D. A. and Lesesne, H. R. (1984). Hepatobiliary disease that precedes ulcerative colitis. *J. Clin. Gastroenterol*, **6**, 425–428.
[69] Stockbrugger, R. W., Olsson, R., Jaup, B. and Jensen, J. (1988). Forty-six patients with primary sclerosing cholangitis: radiological bile duct changes in relationship to clinical course and concomitant inflammatory bowel disease. *Hepatogastroenterol*, **35**, 289–294.

[70] Retsky, J. E. and Kraft, S. C., The Extraintestinal manifestations of inflammatory bowel disease. In: Kirsner, J. B. and Shorter, R. Y. Eds., *Inflammatory Bowel Disease*, 4th edn., Baltimore, Williams and Wilkins, 1995, pp. 474–491.

[71] Hyams, J. S. (1994). Extraintestinal manifestations of inflammatory bowel disease in children. *J. Pediatr. Gastroenterol Nutr.*, **19**, 7–21.

[72] De Maria, N., Colantoni, A., Rosenbloom, E. and Van Thiel, D. H. (1996). Ursodeoxycholic acid does not improve the clinical course of primary sclerosing cholangitis over a 2-year period. *Hepatogastroenterol*, **43**, 1472–1479.

[73] Lindor, K. D. (1997). Ursodiol for primary sclerosing cholangitis. Mayo Primary Sclerosing Cholangitis-Ursodeoxycholic Acid Study Group. *NEJM*, **336**, 691–695.

[74] Wiesner, R. H., LaRusso, N. F., Dozois, R. R. and Beaver, S. J. (1986). Peristomal varices after proctocolectomy in patients with primary sclerosing cholangitis. *Gastroenterol*, **90**, 316–322.

[75] Cameron, A. D. and Fone, D. J. (1970). Portal hypertension and bleeding ileal varices after colectomy and ileostomy for chronic ulcerative colitis. *Gut*, **11**, 755–759.

[76] Kartheuser, A. H., Dozois, R. R., LaRusso, N. F., Wiesner, R. H., Ilstrup, D. M. and Schleck, C. D. (1996). Comparison of surgical treatment of ulcerative colitis associated with primary sclerosing cholangitis: ileal pouch-anal anastomosis *versus* Brooke ileostomy. *Mayo. Clin. Proc.*, **71**, 748–756.

[77] Mikkola, K., Kiviluoto, T., Riihela, M., Taavitsainen, M. and Jarvinen, H. J. (1995). Liver involvement and its course in patients operated on for ulcerative colitis. *Hepatogastroenterol*, **42**, 68–72.

[78] Ludwig, J. (1991). Small-duct primary sclerosing cholangitis. [Review], [40 Refs.] *Semin. Liver Dis.*, **11**, 11–17.

[79] Wee, A. and Ludwig, J. (1985). Pericholangitis in chronic ulcerative colitis: primary sclerosing cholangitis of the small bile ducts?. *Ann. Intern. Med.*, **102**, 581–587.

[80] Perrett, A. D., Higgins, G., Johnston, H. H., Massarella, G. R., Truelove, S. C. and Wrigth, R. (1971). The liver in Crohn's disease. *QJM*, **40**, 187–209.

